# A novel reliability-based regression model to analyze and forecast the severity of COVID-19 patients

**DOI:** 10.1186/s12911-022-01861-2

**Published:** 2022-05-05

**Authors:** Negar Bakhtiarvand, Mehdi Khashei, Mehdi Mahnam, Somayeh Hajiahmadi

**Affiliations:** 1 Department of Industrial and Systems Engineering, Isfahan University of Technology, Isfahan, 84156-83111 Iran; 2 Center for Optimization and Intelligent Decision Making in Healthcare Systems (COID-Health), Isfahan University of Technology, Isfahan, 84156-83111 Iran; 3 Department of Radiology, Isfahan University of Medical Sciences, Isfahan, Iran

**Keywords:** COVID-19, Multiple linear regression (MLR), Forecasting and modeling, Reliability and accuracy, Data analysis, Disease severity

## Abstract

****Background**:**

Coronavirus outbreak (SARS-CoV-2) has become a serious threat to human society all around the world. Due to the rapid rate of disease outbreaks and the severe shortages of medical resources, predicting COVID-19 disease severity continues to be a challenge for healthcare systems. Accurate prediction of severe patients plays a vital role in determining treatment priorities, effective management of medical facilities, and reducing the number of deaths. Various methods have been used in the literature to predict the severity prognosis of COVID-19 patients. Despite the different appearance of the methods, they all aim to achieve generalizable results by increasing the accuracy and reducing the errors of predictions. In other words, accuracy is considered the only effective factor in the generalizability of models. In addition to accuracy, reliability and consistency of results are other critical factors that must be considered to yield generalizable medical predictions. Since the role of reliability in medical decisions is significant, upgrading reliable medical data-driven models requires more attention.

****Methods**:**

This paper presents a new modeling technique to specify and maximize the reliability of results in predicting the severity prognosis of COVID-19 patients. We use the well-known classic regression as the basic model to implement our proposed procedure on it. To assess the performance of the proposed model, it has been applied to predict the severity prognosis of COVID-19 by using a dataset including clinical information of 46 COVID-19 patients. The dataset consists of two types of patients’ outcomes including mild (discharge) and severe (ICU or death). To measure the efficiency of the proposed model, we compare the accuracy of the proposed model to the classic regression model.

****Results**:**

The proposed reliability-based regression model, by achieving 98.6% sensitivity, 88.2% specificity, and 93.10% accuracy, has better performance than classic accuracy-based regression model with 95.7% sensitivity, 85.5% specificity, and 90.3% accuracy. Also, graphical analysis of ROC curve showed AUC 0.93 (95% CI 0.88–0.98) and AUC 0.90 (95% CI 0.85–0.96) for classic regression models, respectively.

****Conclusions**:**

Maximizing reliability in the medical forecasting models can lead to more generalizable and accurate results. The competitive results indicate that the proposed reliability-based regression model has higher performance in predicting the deterioration of COVID-19 patients compared to the classic accuracy-based regression model. The proposed framework can be used as a suitable alternative for the traditional regression method to improve the decision-making and triage processes of COVID-19 patients.

## Background

COVID-19 which was initially emerged from Wuhan, China in December 2019 has spread rapidly all around the world and has caused serious challenges for public health, economic and social activities. COVID-19 pandemic has put considerable pressure on governments and healthcare systems. In this crisis situation, predicting the disease severity of arriving patients can play a fundamental role in saving more lives. It helps treatment teams to prioritize patients who are more likely to have an acute condition (ICU admission or death), which in turn accelerates the triage and healing processes, reduces the number of deaths, and causes more efficient resource management. Patient characteristics including clinical data and computed tomography (CT) imaging have been studied by researchers to achieve precise predictions about COVID-19 severity. Gallo Marin et al. [15] have surveyed useful features in predicting the severity of COVID-19 disease. The factors include patients’ age, comorbidities, immune response, radiographic findings, laboratory markers, and indicators of organ dysfunction. Francone et al. [14] have studied CT scores and laboratory findings of SARS-CoV-2 patients. The results have shown that CT score has a critical role in forecasting the outcome of patients and there is a high correlation between this score and laboratory findings. Rokni et al. [36] have compared clinical, para-clinical, and laboratory findings between survived and deceased COVID-19 patients by using an independent sample T-test. The results show that elevated neutrophil to lymphocyte ratio (NLR), platelet to lymphocyte ratio (PLR), and systematic immune-inflammation (SII) can be considered as prognostic and risk stratifying factors of the severe form of COVID-19. Zhang et al. [48] have compared clinical, laboratory, and CT findings between the survived and deceased groups of patients. Their results have shown that older age, comorbidities such as diabetes and emphysema, and higher CRP and NLRs increase the risk of death in Covid-19 patients. The literature of forecasting in COVID-19, specifically for disease severity, shows a great interest to apply model-based approaches in different forms. In general, these models can be categorized into two main categories of analytical and predictical approaches. In the analytical approaches, the final goal is to yield a valid model for analyzing the underlying relationships between the target variable to the explanatory variable(s). While the main goal of the predictical approaches is to predict the target variable. Both of these categories are beneficial in their domain and have been applied in a wide range of applications, successfully.

Statistical and intelligent models are two main classes of methods that have been used in this field. The use of statistical techniques is a common approach to develop COVID-19 severity, prediction models. Regression models are among the most commonly used statistical methods in medical predictions. Different forms of regression models such as classic regression, logit regression, Cox regression, and least absolute shrinkage and selection operator (LASSO), etc. are among the most important statistical methods that have been used frequently in COVID-19 severity prediction researches. Hajiahmadi et al. [16] have used a multivariate regression model to show the usefulness of chest severity score (CSS) in predicting ICU admission and mortality. Homayounieh et al. [18] have applied a multiple logistic regression model to show the superiority of the radionics from non-contrast chest CT over the radiologists’ estimation in predicting the outcome of COVID-19. Huang et al. [19] have shown that clinical attributes including underlying diseases, increased respiratory rate, elevated C-reactive protein (CRP), and lactate dehydrogenase (LDH) have a significant correlation with the progress severity of COVID-19. The obtained results also indicate that elevated lactate dehydrogenase can be used as an effective feature to differentiate severe cases from mild patients. They have utilized single-factor and multivariate logistic regression models as prediction methods. Zhou et al. [52] have studied Demographics, symptoms, comorbidities, and temporal changes of laboratory results, CT features and severity scores for recovered and deceased groups by employing Mann-Whitney U test and the logistic regression model. Xiao et al. [44] have applied univariable and multivariable logistic regression models by using demographic, clinical, laboratory, and radiological data of COVID-19 patients. Their findings show that maximum CT score (>11) and chronic obstructive pulmonary disease (COPD) are critical features that affect the deterioration of COVID-19 patients. Shi et al. [37] have employed a LASS logistic regression model to predict the severity of COVID-19 disease based on clinical and radiological findings of patients at admission. Wei et al. [42] have applied the value of CT texture analysis and clinical parameters to predict severe COVID-19 patients. They first have performed a minimum redundancy and maximum relevance (MRMR) method to feature selection and secondly have applied selected features as independent variables in a multivariate logistic regression framework. Zhang et al. [48] have used univariable and multivariable logistic regression models to determine the risk factors of COVID-19 severity including age, white blood cell count, neutrophil, glomerular filtration rate, and myoglobin. A scoring system has been built according to the hazard ratio of each selected feature and the system has been used to predict severe COVID-19 patients. Chen et al. [8] have determined risk factors on fetal status for COVID-19 hospitalized patients by employing multivariate Cox regression analysis. The risk factors include advanced age, dyspnea, coronary heart disease (CHD), cerebrovascular disease (CVD), and elevated levels of procalcitonin (PCT) and aspartate aminotransferase (AST). Bi et al. [6] have studied factors of coagulation function in COVID-19 patients. Their results show that fibrinogen-to-Albumin Ratio (FAR) and platelet count (PLT) are two important features in predicting the progression of severe disease by applying a multivariate Cox analysis. Zhou et al. [53] have used the LASSO regression model to determine effective factors on COVID-19 severity including body temperature at admission, cough, dyspnea, hypertension, cardiovascular disease, chronic liver disease, and chronic kidney disease. They have utilized a multivariable logistic regression to achieve COVID-19 severity predictions. Dong et al. [12] have employed Cox regression models to identify high-risk features in COVID-19 severity. The features which include comorbidities, advanced age, reduced lymphocyte count, and higher lactate dehydrogenase at presentation are applied to make a scoring forecasting model.McRae et al. [30] have used logistic regression model by using different attributes including CRP, N-terminus pro B type natriuretic peptide (NT-proBNP), myoglobin (MYO), D-dimer, PCT, creatine kinase-myocardial band (CK-MB), and, cardiac troponin I (cTnI) to determine COVID-19 severity. Zhang et al. [49] have employed the Cox regression method to forecast recovery in adult hospitalized COVID-19 patients in the short term.

As well as statistical models that are useful tools in modeling and analysis, machine learning and artificial intelligence methods have attracted a great deal of attention in the field of COVID-19 severity prediction. Li et al. [27] have shown the effectiveness of laboratory tests and CT data to predict severe cases by employing a machine learning approach based on the random forest approach. Matos et al. [29] have provided a prediction of short-term outcomes in COVID-19 patients. They have shown that the volume of disease on CT scans and clinical attributes are useful to predict short-term outcomes. They have applied lymphocyte percentage and C-reactive protein to predict the volume of disease on CT scans. Different classification methods have been employed in their work including generalized linear model (GLM), penalized binominal regression (PBR), conditional inference trees (CIT), and support vector machine with the linear kernel (SVL). Zhou et al. [51] have examined a set of clinical factors including oxygenation index, basophil counts aspartate aminotransferase, gender, magnesium, gamma-glutamyl transpeptidase, platelet counts, activated partial thromboplastin time, oxygen saturation, body temperature, and days after symptom onset to achieve a predict of COVID-19 disease development. They have used a genetic algorithm (GA) as a feature selection method as well as support vector machine (SVM) model to make the predictions. Yan et al. [46] have proposed an XGBoost machine-learning model to predict critically ill patients by using lactic dehydrogenase (LDH), lymphocyte, and High-sensitivity C-reactive protein (hsCRP) factors. Ning et al. [31] have prepared a deep learning approach to predict COVID-19 patient outcomes by using CT images and 130 clinical features including biochemical and cellular analyses of blood and urine samples. Bai et al. [5] have used clinical, laboratory, and CT data to predict COVID-19 malignant progression by utilizing different approaches including logistic regression model, linear discriminant analysis (LDA), SVM, Multilayer perceptron (MLP), and long short term memory (LSTM) methods. They have proposed a machine-learning-based model for severity prediction which outperforms the logistic regression model. Cheng et al. [9] have applied a random forest (RF) model to forecast ICU Transfer within 24 h for COVID-19 patients who are hospitalized. Al-Najjar and Al-Rousan [2] have studied the effect of various variables including sex, birth year, country, region, group, infection reason, and confirmed date on the outcome (death or survival) of a set of COVID-19 patients by applying neural networks. Their results show that infection reason, confirmation date, and region are the most crucial factors in deceased cases while region, birth year, and confirmation date are the most effective features in survived patients. Moreover, the least effective factors in deceased cases include sex and group where the least important factors in survived patients are infection reason and country. Several researches carried out in this field have been summarized in Table 1.

**Table 1:** Recent studies on predicting the severity of Covid-19 patients

Author/[Ref.]	Scope	Attributes	Methods	Performance	Size	Country
Zhang et al. [48]	Severity of COVID-19	Clinical and laboratory variables	Univariable and multivariable logistic regression models	AUC=0.906	80	China
Hajiahmadi et al. [16]	ICU and death	CT severity score	Logistic regression model	AUC=0.764	192	Iran
Homayounieh et al. [18]	ICU and death	Interpretation of radiologists, clinical variables, lung radiomics	Multiple logistic regression model	AUC =0.84 (for ICU admission)	315	Iran
Huang et al. [19]	Severe cases	Clinical and laboratory data	Single-factor and multivariate logistic regression	AUC = 0.985 (95% CI 0.968–1.00)	125	China
Zhou et al. [52]	Severe cases	clinical, laboratory, and CT data	Multivariable logistic regression	AUC =0.952	134	China
Xiao et al. [44]	Severe illness	Demographic, clinical, laboratory, and radiological data	Univariable and multivariable logistic regression models	AUC= 0.861 (95% CI 0.811–0.902)	243	China
Wei et al. [10]	Common and severe patients	Clinical and CT data	Multivariate logistic regression	AUC=0.95	81	China
Dong et al. [12]	Survival	Clinical and laboratory findungs	Multivariable Cox regression model	AUC= 0.922 (14 days) AUC= 0.881 (21 days)	628	China
Bai et al. [5]	Severity of disease	Clinical, laboratory, and CT data	Logistic regression model, LDA, SVM, MLP and LSTM	AUC=0.954	133	China
Al-Najjar and Al-Rousan [2]	Recovered and death cases	Sex, birth year, country, region, group, infection reason, and confirmed date on the outcome	Neural network	Accuracy=0.938 Accuracy=0.995	1308	South Korea
Li et al. [27]	Severe cases	CT scan data and clinical biochemical attributes	Machine-learning models	AUC =0.93	46	China
Matos et al. [29]	Mechanical ventilation, death	CT scan and clinical attributes	GLM, PBR, CIT, and SVL	AUC =0.92	106	Italy
Ning et al. [31]	Negative, mild, and severe cases	CT images and clinical features	CNN, DNNs, and PLR	AUC = 0.944 (negative) AUC = 0.860 (mild) AUC = 0.884 (severe)	1521	China
Zhou et al. [51]	Severe cases	Clinical factors	GA and SVM	Accuracy: over 0.94 Accuracy= 0.80	144 25	China
Yan et al. [46]	Survival for severe cases	Clinical data	XGBoost algorithm	Accuracy=0.93	375	China
Shi et al. [37]	Severe cases	Clinical and radiological findings	LASSO logistic regression	AUC= 0.890	196	China
Bi et al. [6]	Severe illness	Fibrinogen-to-albumin ratio (FAR) and platelet count (PLT)	Multivariate cox analysis	AUC=0.754	113	China
Zhou et al. [53]	Severe cases	Body temperature, cough, dyspnea, hypertension, cardiovascular disease, chronic liver disease, and chronic kidney disease	Multivariable logistic regression	AUC= 0.862 (95% CI 0.801–0.925)	366	China
Cheng et al. [9]	ICU transfer	Signs, nursing assessments, laboratory features and electrocardiograms	Random forest	AUC= 0.799 (95% CI 0.752–0.846)	1987	USA
McRae et al. [30]	Death	CRP, NT-proBNP, MYO, D-dimer, PCT, CK-MB, cTnI	Logistic regression model	AUC= 0.94 (95% CI 0.89–0.99)	160	China

Despite the different appearance of Covid-19 severity prediction models, they all have been developed based on logic and common idea. The idea is that maximizing accuracy in a predefined training dataset (known patients) leads to higher generalizability in the unknown testing dataset (unseen samples). This means that the accuracy of results is considered as the only factor to determine the generalizability of forecasting models. Although it is a reasonable and frequent approach, it is not the only effective factor in making generalizable predictions. Undoubtedly, the consistency or stability of models’ performance is also important to make proper decisions. In other words, a model with less variety will have more reliability which is an important issue in making medical forecasts. Increasing the reliability of medical forecasting models increases the survival chance of the patients and makes the treatment process more cost-efficient and time-efficient. In other words, the reliability of accuracy is another critical factor in yielding more generalizable and confident medical results that have not been taken into consideration in the modeling processes. In general, increasing the reliability of medical results is usually examined through reducing errors in laboratory tests, errors of equipment, and human error. In this paper, we propose a reliability-based approach to maximize the reliability of accuracy instead of accuracy and achieve more confident predictions in the severity prognosis of COVID-19 patients. In fact, developing data-driven prediction approaches to maximize the reliability of the models’ performance has been mainly ignored in the literature.

The main idea of this paper is to quantify the changes in the accuracy of models’ performance and minimize these changes to maximize reliability. In addition, the variety in this approach has been measured by the variance function. This implies that as the changes in the performance accuracy of the model decrease in the training or validation set, the reliability of the results for the unseen test set increases. To achieve this goal, the classic regression model is chosen to implement the proposed approach. This model has been used to predict various applications in medicine, engineering, energy, finance, management, environment, etc., in the literature. We briefly describe recent researche in a wide range of applications to show the importance and efficiency of this method.

In medicine, Rath et al. [35] applied the multiple linear regression techniques (MLR) to predict the next day’s trend in the active cases of coronavirus disease in Odisha and India. These models acquired remarkable accuracy in COVID-19 recognition. Tang et al. [40] established the MLR model using radial artery pulse wave characteristic parameters to assess vascular aging. Huang et al. [20] presented a K-means-based multiple linear regression model to predict new local Chronic Obstructive Pulmonary Disease hospitalizations number per week with major air pollutants. This prediction model between Chronic Obstructive Pulmonary Disease and air pollutants helps early identification, individualized interventions to slow disease progression, and reduces medical expenditures. The mean absolute percentage error (MAPE) was used to evaluate the model efficiency.

In engineering, Ciulla and D’Amico [10] developed the MLR method to determine the thermal heating or cooling energy demand of a generic building in any weather condition. The promising results justify the use of MLR as an alternative method, issuing an immediate and straightforward tool that can solve a complex problem like building energy balance. Park et al. [33] predicted the large-scale ground source heat pump system’s hourly heating performance with satisfactory accuracy by the MLR and artificial neural network (ANN) models. This research demonstrated the advantage of MLR for the interpretation of the quantitative analysis of performance influencing factors for the ground source heat pump system’s performance. In energy, Çerçi and Hürdoğan [7] designed the MLR and ANN models to estimate the dry-bulb temperature and absolute humidity values of the process air coming out of the process outlet of a desiccant wheel. The coefficient of determination (R2), Mean Absolute Error (MAE), and Root Mean Square Error (RMSE) criteria were used to determine the consistency of the results obtained from different models to the manufacturer’s data. Khemet and Richman [23] predicted the quantity of air leakage in houses based on variables including building geometry, building materials, building age, and local climate by using the MLR model. Siavash et al. [38] predicted the turbine power curve and rotor speed for the small wind turbine equipped with a wide range of duct opening angles at any wind speed using the MLR and ANN models. Four MLR models in different shapes and a multi-layer perceptron neural network is presented to estimate the power and rotor angular speed of a wind turbine equipped with a variable shroud. The accuracy of prediction models was presented using RMSE and R2 for both the ANN and MLR models. In agriculture, Abrougui et al. [1] evaluated the MLRs and ANNs to predict organic potato crop yield by using tillage systems and soil properties. The results showed that the MLR model estimated crop yield more accurately than the ANN model. Lee et al. [25] used the MLR model to estimate the soil moisture’s spatial distribution in South Korea. The coefficients of the MLR model were estimated seasonally considering five days of preceding precipitation. Xie et al. [45] conducted the MLR and random forest regression (RFR) models to estimate soil amylase and urease activities in long-term coastal reclaimed land. Pahlavan-Rad et al. [32] compared the MLR and the RFR models for predicting soil infiltration rates in a dry flood plain of eastern Iran. The model RMSE and MAE evaluation metrics were similar between models. In environment, Stoichev et al. [39] used an innovative MLR model to evaluate metal/metalloid contamination in a coastal lagoon’s surface sediments. Yuchi et al. [47] used the MLR and RFR to model indoor air pollution with 87 potential predictor variables from outdoor monitoring data, questionnaires, home assessments, and geographic data sets. Tang et al. [41] developed the MLR and support vector machine algorithms to predict biodegradation rate as a significant process for removing organic chemicals from water, soil, and sediment environments. Amoozad-Khalili et al. [3] investigated the relationship between input costs and the income of wheat production in mechanized and semi-mechanized systems using various MLR models. In finance, Cogoljević et al. [11] applied the MLR analysis to determine how consumer price index, monetary aggregates, discount rate, and exchange rate affect inflation. Based on the results, one can observe an acceptable correlation, which means there is a strong correlation between reals and estimated values.

Moreover, recently, Zheng et al. [50] by using the MLR techniques examined how process conditions (r.g., temperature and duration) and feedstock properties affect the product characteristics. According to the R2 and RMSE, the developed MLR model had an excellent quantitative determination of hydrothermal carbonization properties with high accuracy. Kern et al. [21] applied many MLR models for the prediction of dry matter during curd treatment. The best models were selected based on Akaike’s information criterion (AICc), R2, and most parsimonious construction to describe the data set. Kusano et al. [24] developed the MLR analysis to predict the tensile properties using several microstructural features for selective laser melted and post heat-treated. The model showed good accuracy for predicting. Rahbari et al. [34] provided the MLR model as a conceptually simple and computationally efficient way of computing thermodynamic derivatives for multicomponent systems analysis. Hoang [17] proposed the MLR and ANN models for estimating the punching shear capacity of steel fiber reinforced concrete (SFRC) slabs. Experimental results show that MLR can deliver prediction outcomes better than those of ANN and empirical design equations. Therefore, MLR can be a promising alternative to assist structural engineers in designing structures.

There are two main reasons to employ the classical linear regression model for implementing the proposed reliability-based approach. First, the classical linear regression with low complexity eliminates the effect of other features such as the impact of design and complexity of models on generalization power, and the increase in model generalizability only originates from increasing in the reliability. Second, the initial purpose of this paper is to analyze the severity of Covid-19 in addition to forecasting it. Therefore, the state-of-the-art models which have not the capability to analyze the relationship between the variables have not been considered and the regression model which is considered as a popular method for analysis purposes is chosen.

All MLR models in the literature have identical thinking on the method of modeling. The logic of creating such models is to maximize the performance accuracy of the training data to achieve maximum accuracy in the test data or the model’s generalization ability. Accordingly, the generalization ability in this type of model is considered only related to performance accuracy. Although the accuracy is one of the most important factors affecting the model’s generalization ability, it is not the unique factor explaining how to change the model’s generalization ability. It seems that one of the other factors affecting the generalization ability of the model is the degree of confidence in performance accuracy, or in other words, changes in performance accuracy in the face of different conditions that are not considered in the conventional thinking of MLR modeling. In fact, he performance basis in conventional regression modeling is based on the assumption that maximum accuracy in inaccessible data is obtained from models with the least amount of error in modeling available data. In this type of regression modeling, in order to maximize the generalization ability of simulations, which are the main factor influencing the quality of decisions made in real-world problems, the principle of maximization of the accuracy of available historical data is used. However, in this type of modeling process, the model’s reliability and its results have not been considered. On the other hand, the generalization capability of a model is simultaneously dependent on the accuracy of the model and the reliability level of the accuracy. In this paper, a new methodology is proposed for multiple linear regression modeling; in contrast to traditionally developed models, the constructed models’ reliability is maximized instead of its accuracy.

To show the effectiveness of the proposed Reliability-based regression (RbR) model, it has been applied to predict the severity of COVID-19 disease. A dataset including clinical findings of 46 patients with COVID-19 symptoms is studied and the severe cases are predicted by applying the proposed framework. The results indicate the superiority of the proposed RbR model over the classic regression model.

The remainder of this paper is organized as follows: In the next section, the concepts and formulation of the proposed RbR model are presented. In "Results and discussion" section, the dataset is described and the proposed RbR model is applied to predict disease severity of COVID-19 patients in mentioned dataset and its performance is compared with the traditional regression model. Finally, and in the last section, we represent conclusions.

## Method

Traditional modeling approaches in medical predictions all have been developed based on a common theory, which indicates that accuracy in the training set is supposed as the only effective factor on the generalizability of models. However, models’ generalizability as an important factor in applying the model to solve real-world problems depends on both the accuracy and reliability of results. In fact, another way to enhance the generalizability of disease diagnosis models is increasing the reliability of the results and the reproducibility of the models’ performance. Given the importance of achieving reliable results in the process of diagnosis and treatment of diseases, in this study, a new Reliability-based regression (RbR) model has been developed to maximize reliability rather than accuracy in diagnostic methods. The basic concept of the presented model is quantifying the fluctuations of performance in the training data or a portion of it (validation data) and minimizing these fluctuations to ensure higher reliability and generalizability in the test data. Therefore, in the first step, the data is divided into the training and testing data, and next a part of the training data is selected for validation data. To achieve the maximum reliability, the unknown parameters in the proposed approach are calculated in such a manner that the fluctuations of the model’s performance are minimized for the validation data.

In the following, first, the traditional multiple regression model, as a well-known statistical technique in medical applications, is briefly described and then the procedure of the suggested reliability-based regression template is explained in detail.

Multiple Linear Regression is broadly used in medical prediction researches, especially in modeling and analysis linear relationships between one output variable such as disease severity and one or several input variables such as patients’ attributes. A linear regression model can be shown as follows:

1 $$\begin{aligned} \begin{aligned} Y_i=\beta _1+\beta _2 X_{2i}+\beta _3 X_{3i}+\cdots +\beta _k X_{ki}+u_i \quad \quad i=1,2,\ldots , N \end{aligned} \end{aligned}$$

where *Y* represents the output variable, $X_1, X_2, \ldots , X_k$ are the output explanatory variables, $\beta _1$ is the intercept of the regression line, $\beta _2$ to $\beta _k$ are regression coefficients, (slopes), *u* is the residual term, and *N* is the number of samples. The operation of the ordinary least square (OLS) technique which is used to estimate unknown parameters of the above formula is based on minimizing error (the difference between actual and predicted values) squares. In other words, OLS is an accuracy-based technique. In contrast, the procedure of our proposed model is based on this key idea that minimizing the variation of errors’ squares, results in maximizing the reliability of predictions. To perform this model, first, a section of the training data set is considered as the validation data set. In this paper, the accuracy, as sum of squared errors, for the training data as well as training data plus each data of the validation is determined as follows:

2 $$\begin{aligned} \begin{aligned} \sum _{t=1}^N {\hat{u}}_{0t}^2 = \sum _{t=1}^N \left( Y_t - {\hat{\beta }}_{01} X_{1t} - {\hat{\beta }}_{02} X_{2t} - \cdots -{\hat{\beta }}_{0k} X_{kt}\right) ^2 = \sum _{t=1}^N \left( Y_t - \sum _{j=1}^k {\hat{\beta }}_{0j} X_{jt}\right) ^2 \end{aligned} \end{aligned}$$

and in the same manner, for each member of the validation data set:

3 $$\begin{array}{*{20}c} {\sum\limits_{{t = 1}}^{{N + 1}} {\hat{u}_{{1t}}^{2} } = \sum\limits_{{t = 1}}^{{N + 1}} {\left( {Y_{t} - \hat{\beta }_{{11}} X_{{1t}} - \cdots - \hat{\beta }_{{1k}} X_{{kt}} } \right)^{2} } = \sum\limits_{{t = 1}}^{{N + 1}} {\left( {Y_{t} - \sum\limits_{{j = 1}}^{k} {\hat{\beta }_{{1j}} } X_{{jt}} } \right)^{2} } } \\ {\sum\limits_{{t = 1}}^{{N + 2}} {\hat{u}_{{2t}}^{2} } = \sum\limits_{{t = 1}}^{{N + 2}} {\left( {Y_{t} - \hat{\beta }_{{21}} X_{{1t}} - \cdots - \hat{\beta }_{{2k}} X_{{kt}} } \right)^{2} } = \sum\limits_{{t = 1}}^{{N + 2}} {\left( {Y_{t} - \sum\limits_{{j = 1}}^{k} {\hat{\beta }_{{2j}} } X_{{jt}} } \right)^{2} } } \\ { \cdots \cdots \cdots \cdots } \\ {\sum\limits_{{t = 1}}^{{N + n}} {\hat{u}_{{nt}}^{2} } = \sum\limits_{{t = 1}}^{{N + n}} {\left( {Y_{t} - \hat{\beta }_{{n1}} X_{{1t}} - \cdots - \hat{\beta }_{{nk}} X_{{kt}} } \right)^{2} } = \sum\limits_{{t = 1}}^{{N + n}} {\left( {Y_{t} - \sum\limits_{{j = 1}}^{k} {\hat{\beta }_{{nj}} } X_{{jt}} } \right)^{2} } } \\ \end{array}$$

where $\sum _{t=1}^{N+i} {\hat{u}}_{it}^2$ for $i=0,1,\ldots ,n$ and $t=1,2,\ldots ,N+i$ are the residual sum of squares (RSS), and is the size *n* of validation dataset. To determine the optimal value of unknown parameters in each data point, $\beta _{ij} \quad i=0,1,\ldots ,n \quad j=1,2,\ldots ,k$, they are determined in such a way that $\sum {\hat{u}}_{it}^2$ is minimized [13, 22]. This is performed by differentiating each equation partially with respect to parameters in each data point and setting the results to zero. The process yields *k* simultaneous equations in *k* unknowns, for each data point, as follows. For the training data:

4 $$\begin{array}{*{20}c} {\hat{\beta }_{{01}} \sum\limits_{{t = 1}}^{N} {X_{{1t}}^{2} } + \hat{\beta }_{{02}} \sum\limits_{{t = 1}}^{N} {X_{{1t}} } X_{{2t}} + \hat{\beta }_{{03}} \sum\limits_{{t = 1}}^{N} {X_{{1t}} } X_{{3t}} + \cdots + \hat{\beta }_{{0k}} \sum\limits_{{t = 1}}^{N} {X_{{1t}} } X_{{kt}} = \sum\limits_{{t = 1}}^{N} {X_{{1t}} } Y_{t} } \\ {\hat{\beta }_{{01}} \sum\limits_{{t = 1}}^{N} {X_{{2t}} } X_{{1t}} + \hat{\beta }_{{02}} \sum\limits_{{t = 1}}^{N} {X_{{2t}}^{2} } + \hat{\beta }_{{03}} \sum\limits_{{t = 1}}^{N} {X_{{2t}} } X_{{3t}} + \cdots + \hat{\beta }_{{0k}} \sum\limits_{{t = 1}}^{N} {X_{{2t}} } X_{{kt}} = \sum\limits_{{t = 1}}^{N} {X_{{2t}} } Y_{t} } \\ {\hat{\beta }_{{01}} \sum\limits_{{t = 1}}^{N} {X_{{3t}} } X_{{1t}} + \hat{\beta }_{{02}} \sum\limits_{{t = 1}}^{N} {X_{{3t}} } X_{{2t}} + \hat{\beta }_{{03}} \sum\limits_{{t = 1}}^{N} {X_{{3t}}^{2} } + \cdots + \hat{\beta }_{{0k}} \sum\limits_{{t = 1}}^{N} {X_{{3t}} } X_{{kt}} = \sum\limits_{{t = 1}}^{N} {X_{{3t}} } Y_{t} } \\ { \cdots \cdots \cdots \cdots } \\ {\hat{\beta }_{{01}} \sum\limits_{{t = 1}}^{N} {X_{{kt}} } X_{{1t}} + \hat{\beta }_{{02}} \sum\limits_{{t = 1}}^{N} {X_{{2t}} } X_{{kt}} + \hat{\beta }_{{03}} \sum\limits_{{t = 1}}^{N} {X_{{3t}} } X_{{kt}} + \cdots + \hat{\beta }_{{0k}} \sum\limits_{{t = 1}}^{N} {X_{{kt}}^{2} } = \sum\limits_{{t = 1}}^{N} {X_{{kt}} } Y_{t} } \\ \end{array}$$

and in the same way, for the first data of the validation data set:

5 $$\begin{array}{*{20}c}{\hat{\beta }}_{11} \sum _{t=1}^{N+1} X_{1t}^2 + {\hat{\beta }}_{12} \sum _{t=1}^{N+1} X_{1t} X_{2t} + \cdots + {\hat{\beta }}_{1k} \sum _{t=1}^{N+1} X_{1t} X_{kt} = \sum _{t=1}^{N+1} X_{1t} Y_{t}\\{\hat{\beta }}_{11} \sum _{t=1}^{N+1} X_{2t} X_{1t} + {\hat{\beta }}_{12} \sum _{t=1}^{N+1} X_{2t}^2 + \cdots + {\hat{\beta }}_{1k} \sum _{t=1}^{N+1} X_{2t} X_{kt} = \sum _{t=1}^{N+1} X_{2t} Y_{t}\\{\hat{\beta }}_{11} \sum _{t=1}^{N+1} X_{3t} X_{1t} + {\hat{\beta }}_{12} \sum _{t=1}^{N+1} X_{3t} X_{2t} + \cdots + {\hat{\beta }}_{1k} \sum _{t=1}^{N+1} X_{3t} X_{kt} = \sum _{t=1}^{N+1} X_{3t} Y_{t}\\ \cdots \cdots \cdots \cdots \\{\hat{\beta }}_{11} \sum _{t=1}^{N+1} X_{kt} X_{1t} + {\hat{\beta }}_{12} \sum _{t=1}^{N+1} X_{kt} X_{2t} + \cdots + {\hat{\beta }}_{1k} \sum _{t=1}^{N+1} X_{kt}^2 = \sum _{t=1}^{N+1} X_{kt} Y_{t}\\ \end{array}$$

For the last data of the validation dataset, we have:

6 $$\begin{array}{*{20}c}{\hat{\beta }}_{n1} \sum _{t=1}^{N+n} X_{1t}^2 + {\hat{\beta }}_{n2} \sum _{t=1}^{N+n} X_{1t} X_{2t} + \cdots + {\hat{\beta }}_{nk} \sum _{t=1}^{N+n} X_{1t} X_{kt} = \sum _{t=1}^{N+n1} X_{1t} Y_{t}\\{\hat{\beta }}_{n1} \sum _{t=1}^{N+1} X_{2t} X_{1t} + {\hat{\beta }}_{n2} \sum _{t=1}^{N+n} X_{2t}^2 + \cdots + {\hat{\beta }}_{nk} \sum _{t=1}^{N+n} X_{2t} X_{kt} = \sum _{t=1}^{N+n1} X_{2t} Y_{t}\\{\hat{\beta }}_{n1} \sum _{t=1}^{N+n} X_{3t} X_{1t} + {\hat{\beta }}_{n2} \sum _{t=1}^{N+n} X_{3t} X_{2t} + \cdots + {\hat{\beta }}_{nk} \sum _{t=1}^{N+n} X_{3t} X_{kt} = \sum _{t=1}^{N+n} X_{3t} Y_{t}\\ \cdots \cdots \cdots \cdots \\{\hat{\beta }}_{n1} \sum _{t=1}^{N+n} X_{kt} X_{1t} + {\hat{\beta }}_{n2} \sum _{t=1}^{N+n} X_{2t} X_{kt} X_{2t} + \cdots + {\hat{\beta }}_{nk} \sum _{t=1}^{N+n} X_{kt}^2 = \sum _{t=1}^{N+n} X_{kt} Y_{t}\\ \end{array}$$

To construct the RbR model with the minimum deviation of squared errors in validation samples, the unknown parameters of all accuracy-based regression lines must be equal. Thus, we have:

7 $$\begin{array}{*{20}c}{\hat{\beta }}_{ij} ={\hat{\beta }}_{i' j} \qquad \forall i, i' = 0,1,2,\ldots ,n, \quad \forall j=1,2,\ldots , k\\{\hat{\beta }}e_{j} = {\hat{\beta }}_{ij} \qquad \forall i= 0,1,2,\ldots ,n, \quad \quad \forall j=1,2,\ldots , k\\ \end{array}$$

where, ${\hat{\beta }}e_{j}$ is the *j*th parameter of the RbR model. Eventually, Eqs. (4–6) could be shown as follows:

8 $$\begin{array}{*{20}c}{\hat{\beta }}e_{1} \sum _{i=0}^{n}\sum _{t=1}^{N+i} X_{1t}^2 + {\hat{\beta }}e_{2} \sum _{i=0}^{n}\sum _{t=1}^{N+i} X_{1t} X_{2t} + \cdots + {\hat{\beta }}e_{k} \sum _{i=0}^{n}\sum _{t=1}^{N+i} X_{1t} X_{kt} = \sum _{i=0}^{n}\sum _{t=1}^{N+i} X_{1t} Y_{t}\\{\hat{\beta }}e_{1} \sum _{i=0}^{n}\sum _{t=1}^{N+i} X_{2t} X_{1t} + {\hat{\beta }}e_{2} \sum _{i=0}^{n}\sum _{t=1}^{N+i} X_{2t}^2 + \cdots + {\hat{\beta }}e_{k} \sum _{i=0}^{n}\sum _{t=1}^{N+i} X_{2t} X_{kt} = \sum _{i=0}^{n}\sum _{t=1}^{N+i} X_{2t} Y_{t}\\{\hat{\beta }}e_{1} \sum _{i=0}^{n}\sum _{t=1}^{N+i} X_{3t} X_{1t} + {\hat{\beta }}e_{2} \sum _{i=0}^{n}\sum _{t=1}^{N+i} X_{3t} X_{2t} + \cdots + {\hat{\beta }}e_{k} \sum _{i=0}^{n}\sum _{t=1}^{N+i} X_{3t} X_{kt} = \sum _{i=0}^{n}\sum _{t=1}^{N+i} X_{3t} Y_{t}\\ \cdots \cdots \cdots \cdots \\{\hat{\beta }}e_{1} \sum _{i=0}^{n}\sum _{t=1}^{N+i} X_{kt} X_{1t} + {\hat{\beta }}e_{2} \sum _{i=0}^{n}\sum _{t=1}^{N+i} X_{kt} X_{2t} + \cdots + {\hat{\beta }}e_{k} \sum _{i=0}^{n}\sum _{t=1}^{N+i} X_{kt}^2 = \sum _{i=0}^{n}\sum _{t=1}^{N+i} X_{kt} Y_{t}\\ \end{array}$$

The equations are presented in a matrix format as follows:

9 $$\begin{aligned} \!\!\!\!\!\!\!\!\!\!\! \begin{bmatrix} \sum \limits _{i=0}^{n}\sum \limits _{t=1}^{N+i} X_{1t}^2 & \sum \limits _{i=0}^{n}\sum \limits _{t=1}^{N+i} X_{1t} X_{2t} & \cdots & \sum \limits _{i=0}^{n}\sum \limits _{t=1}^{N+i} X_{1t} X_{kt} \\ \sum \limits _{i=0}^{n}\sum \limits _{t=1}^{N+i} X_{2t} X_{1t} & \sum \limits _{i=0}^{n}\sum \limits _{t=1}^{N+i} X_{2t}^2 & \cdots & \sum \limits _{i=0}^{n}\sum \limits _{t=1}^{N+i} X_{2t} X_{kt} \\ \sum \limits _{i=0}^{n}\sum \limits _{t=1}^{N+i} X_{3t} X_{1t} & \sum \limits _{i=0}^{n}\sum \limits _{t=1}^{N+i} X_{3t} X_{2t} & \cdots & \sum \limits _{i=0}^{n}\sum \limits _{t=1}^{N+i} X_{3t} X_{kt} \\ \cdots & \cdots & \cdots & \cdots \\ \sum \limits _{i=0}^{n}\sum \limits _{t=1}^{N+i} X_{kt} X_{1t} & \sum \limits _{i=0}^{n}\sum \limits _{t=1}^{N+i} X_{kt} X_{2t} & \cdots & \sum \limits _{i=0}^{n}\sum \limits _{t=1}^{N+i} X_{kt}^2 \\ \end{bmatrix} \begin{bmatrix} {\hat{\beta }}e_{1} \\ {\hat{\beta }}e_{2} \\ {\hat{\beta }}e_{3} \\ \cdots \\ {\hat{\beta }}e_{k} \\ \end{bmatrix} = \begin{bmatrix} \sum \limits _{i=0}^{n}\sum \limits _{t=1}^{N+i} X_{1t} Y_{t} \\ \sum \limits _{i=0}^{n}\sum \limits _{t=1}^{N+i} X_{2t} Y_{t} \\ \sum \limits _{i=0}^{n}\sum \limits _{t=1}^{N+i} X_{3t} Y_{t} \\ \cdots \\ \sum \limits _{i=0}^{n}\sum \limits _{t=1}^{N+i} X_{kt} Y_{t} \\ \end{bmatrix} \end{aligned}$$

At last, the unknown parameters of RbR model can be obtained by solving Eq. (9). For instance, in a 3-variable model, the parameters are estimated as follow:

10 $$\begin{aligned} \begin{aligned} {\hat{\beta }}e_{1}= \frac{(A_{22} A_{33} -A_{23}^2) B_1 - (A_2 A_{33}-A_3 A_{23}) B_2 + (A_2 A_{23} - A_3 A_{22}) B_3}{A_1 A_{22} A_{33} - A_1 A_{23}^2 - A_2^2 A_{33} + 2 A_2 A_3 A_23 - A_{22} A_3^2}\\ {\hat{\beta }}e_{2}= \frac{(A_{3} A_{23} -A_{2} A_{33} ) B_1 - (A_1 A_{33}-A_3^2 ) B_2 + (A_2 A_{3} - A_1 A_{23}) B_3}{A_1 A_{22} A_{33} - A_1 A_{23}^2 - A_2^2 A_{33} + 2 A_2 A_3 A_23 - A_{22} A_3^2}\\ {\hat{\beta }}e_{3}= \frac{(A_{2} A_{23} -A_{3} A_{22} ) B_1 - (A_2 A_{3} - A_1 A_{23} ) B_2 + (A_1 A_{22} - A_2^2 ) B_3}{A_1 A_{22} A_{33} - A_1 A_{23}^2 - A_2^2 A_{33} + 2 A_2 A_3 A_23 - A_{22} A_3^2}\\ \end{aligned} \end{aligned}$$

where $A_{j,j'}=\sum _{i=0}^{n}\sum _{t=1}^{N+i} X_{jt} X_{j't}$ for $j, j' = 1,2,\ldots , k$, and $B_{j}=\sum _{i=0}^{n}\sum _{t=1}^{N+i} X_{jt} Y_{t}$ for $j = 1,2,\ldots , k$.

## Results and discussion

In this study, we have applied clinical features of 46 patients of Covid-19 which have been used by Li et al. [27]. There are more than 300 samples in the dataset, each patient with several samples on different days, related to 105 different tests based on clinical reports. The dataset includes 10 severe and 36 mild patients. These patients visited the People’s Hospital of Yicheng City, China, between January 16, 2020, and March 4, 2020, and were diagnosed with Covid-19. The dataset consists of 6 male and 4 female in severe group and 19 male and 17 female in mild group. The mean age of patients is 48.6. In addition, the mean age of patients in the severe and non-severe groups is 56.8 and 46.5, respectively [27]. Due to the large amount of missing data, 28 factors have been omitted and also for some factors that had less missing values, missing data replaced with the mean values. After normalization and data preprocessing, at last, a group of 50 factors has been selected to analyze and predict the severity of Covid-19 patients (output variable) by using the proposed reliability-based regression and classic accuracy-based regression models. Table 2 summarizes the list of independent variables (clinical factors). The download link of this data set is provided in the Availability of Data and Materials section.

**Table 2:** List of independent variables (clinical factors)

Clinical factor	ID	Symbol	Clinical factor	ID	Symbol
Albumin/globulin	A/G	X1	Mean corpuscular hemoglobin	MCH	X26
Albumin	ALB	X2	Mean corpuscular-hemoglobin concentration	MCHC	X27
Alkaline phosphatase	ALP	X3	Mean corpuscular volume	MCV	X28
Glutamic-pyruvic transaminase	ALT	X4	Absolute value of monocytes	Mono#	X29
Activated partial thromboplastin time	APTT	X5	Percentage of monocytes	Mono%	X30
Glutamic oxalacetic transaminase	AST	X6	mean platelet volume	MPV	X31
Absolute value of basophil	Baso#	X7	Platelet large cell ratio	P-LCR	X32
Percentage of basophils	Baso%	X8	PCT plateletocrit	PCT	X33
Blood urea nitrogen	BUN	X9	Platelet distribution width	PDW	X34
Creatine Kinase Isoenzyme	CK-MB	X10	Blood platelet count	PLT	X35
Creatinine	CREA	X11	Prothrombin time	PT	X36
C-reactive protein	CRP	X12	International normalized ratio	PT-INR	X37
Cystatin C	CysC	X13	Red blood cell count	RBC	X38
Direct bilirubin	D-BIL	X14	CV value of RBC distribution width	RDW-CV	X39
Absolute value of eosinophils	Eos#	X15	SD value of erythrocyte distribution width	RDW-SD	X40
Percentage of eosinophils	Eos%	X16	Sialic acid	SA	X41
Fibrinogen	FIB	X17	Total bile acid	TBA	X42
Gamma-glutamyl transpeptidase	GGT	X18	Total bilirubin	TBIL	X43
Globulin	GLO	X19	Thrombin time	TT	X44
Glucose (fasting)	GLU	X20	Uric acid	UA	X45
Hemoglobin	Hb	X21	${\hat{I}}^2$2-microglobulin	Î$^2$2-MG	X46
Hematocrit value	Hct	X22	Neutrophil absolute value	Neut#	X47
Lactic dehydrogenase	LDH	X23	Neutrophil percentage	Neut%	X48
Lymphocyte absolute value	Lymph#	X24	D-dimer	SF8200_D-Dimer	X49
Percentage of lymphocytes	Lymph%	X25	Cholinesterase	CHE	X50

In the first step, we use the proposed RbR model to analyze the effective variables on disease severity of Covid-19 patients and compute their coefficients using the equations presented in section . The results considering all clinical variables are presented in Table 3. As shown, $R^2$ of the reliability-based model, using all mentioned variables in Table 3, is more than 82%. To interpret the reliability-based regression coefficients and identify the most important risk factors, multicollinearity effects must be eliminated. Moreover, to analyze the relationships between the severity of Covid-19 patients and clinical variables, in each category of highly correlated variables, we keep the variable with the highest correlation to the dependent variable in the model and remove others. The result of performing the RbR model between the severity of Covid-19 patients and selected clinical variables has been shown in Table 4. The results express that the remained clinical features in the model can explain more than 67% of changes in Covid-19 patients. According to the obtained results of the RbR model, the *p*-value is statistically significant (lower than 0.05) for the explanatory variables including X12 (CRP), X13 (CysC), X18(GGT), X21(Hb), X23 (LDH), X25 (Lymph%), and X36 (PT). Table 3 indicates that the largest positive reliability-based coefficients are related to X23 (LDH), X13 (CysC), X36 (PT), X18(GGT), and X12 (CRP), respectively, which means that according to the results of the RbR model the amount of these factors increases in severe cases of Covid-19. Also, the variables X25 (Lymph%), and X21 (Hb) have negative coefficients, which indicates that the amount of these factors decreases in the severe cases of Covid-19 patients. The results are consistent with recent researches, showing elevated levels of LDH, CysC, PT, GGT, and CRP and lower lymphocytes percentage and Hemoglobin in severe cases of Covid-19 patients [4, 26–28, 43]. This means that in the RbR model, in addition to quantifying the changes in the accuracy of the model performance and minimizing these changes to maximize the reliability of results, the effect of influencing factors on the severity of COVID-19 patients is also logical. In the second step, after analyzing the effective variables on the severity of COVID-19 patients, we implement the the reliability-based model to predict COVID-19 disease severity. All of the clinical factors have been used in the prediction model. To make the prediction model, firstly, the data set is divided into a training set (80% of samples) and testing sets (20% of samples). Then, in the next stage, a part of the training data (10%) is applied for validation and obtaining the unknown parameters based on the formulation presented in "Background" section. Due to the specific method of selecting the validation data, and to assure removing all possible data effects on the model’s performance, the procedure has been performed more than 100 times, each time with a different validation dataset.

**Table 3:** Results of RbR model using all clinical factors

Variable	Coefficient	Std. error	t-Statistic	Prob.
Constant	$-0.158417$	1.195739	$-0.132484$	0.8949
X1	0.766133	0.669994	1.143491	0.2556
X2	$-0.588120$	0.369865	$-1.590094$	0.1150
X3	$-0.268347$	0.207036	$-1.296137$	0.1979
X4	$-0.145710$	0.234136	$-0.622331$	0.5351
X5	0.350635	0.224713	1.560370	0.1218
X6	0.124960	0.206834	0.604155	0.5471
X7	0.392064	0.221517	1.769907	0.0798
X9	0.419054	0.302267	1.386370	0.1687
X10	$-0.080296$	0.142200	$-0.564666$	0.5736
X11	$-0.099828$	0.228173	$-0.437509$	0.6627
X12	0.441895	0.324018	1.363796	0.1757
X13	0.782662	0.239895	3.262523	0.0015
X14	$-0.272027$	0.327671	$-0.830183$	0.4084
X15	0.483778	0.200362	2.414519	0.0176
X17	0.140422	0.181415	0.774039	0.4407
X18	0.446554	0.279914	1.595327	0.1138
X19	0.350373	0.507981	0.689735	0.4920
X20	$-0.044969$	0.193640	$-0.232230$	0.8168
X21	$-4.518544$	3.555349	$-1.270914$	0.2067
X22	5.660556	4.708489	1.202202	0.2321
X23	0.282944	0.357990	0.790367	0.4312
X24	$-0.519364$	0.212730	$-2.441429$	0.0164
X26	$-1.317928$	0.937514	$-1.405768$	0.1629
X27	3.450157	2.378494	1.450564	0.1500
X28	$-0.988182$	1.291966	$-0.764867$	0.4462
X29	0.626379	0.410115	1.527327	0.1298
X30	$-0.610015$	0.416580	$-1.464341$	0.1462
X31	0.316610	0.804939	0.393334	0.6949
X32	$-0.281534$	0.816533	$-0.344792$	0.7310
X33	$-0.750111$	0.826578	$-0.907489$	0.3663
X34	0.137479	0.291786	0.471164	0.6385
X35	0.518478	0.863308	0.600571	0.5495
X36	3.783075	6.284011	0.602016	0.5485
X37	$-3.551337$	6.329394	$-0.561086$	0.5760
X38	$-1.789885$	3.175541	$-0.563647$	0.5743
X39	$-0.078335$	0.850181	$-0.092139$	0.9268
X40	0.260570	0.524381	0.496910	0.6203
X41	0.303455	0.300802	1.008819	0.3155
X42	$-0.232581$	0.245079	$-0.949004$	0.3449
X43	0.249179	0.295612	0.842924	0.4013
X44	0.380763	0.267197	1.425027	0.1573
X45	0.192436	0.217392	0.885203	0.3782
X46	$-0.626370$	0.214868	$-2.915135$	0.0044
X47	$-0.451888$	0.458820	$-0.984892$	0.3271
X49	0.075227	0.183322	0.410354	0.6824
X50	$-0.541507$	0.206853	$-2.617839$	0.0102
$${R^2}$$ =0.826801, and adjusted $${R^2}$$ = 0.747130

**Table 4:** Results of RbR model to analyze effective clinical factors on severity of COVID-19 patients

Variable	Coefficient	Std. error	t-Statistic	Prob.
Constant	0.252632	0.140872	1.793350	0.0751
X12	0.386337	0.194240	1.988966	0.0487
X13	0.651536	0.187129	3.481747	0.0007
X18	0.456707	0.160788	2.840435	0.0052
X21	$-0.422841$	0.142370	$-2.970020$	0.0035
X23	0.719013	0.172711	4.163093	0.0001
X25	$-0.693047$	0.147318	$-4.704420$	0.0000
X36	0.466564	0.169386	2.754448	0.0067
${R^2}$ =0.676711, and adjusted ${R^2}$ = 0.660431				

**Fig. 1:**
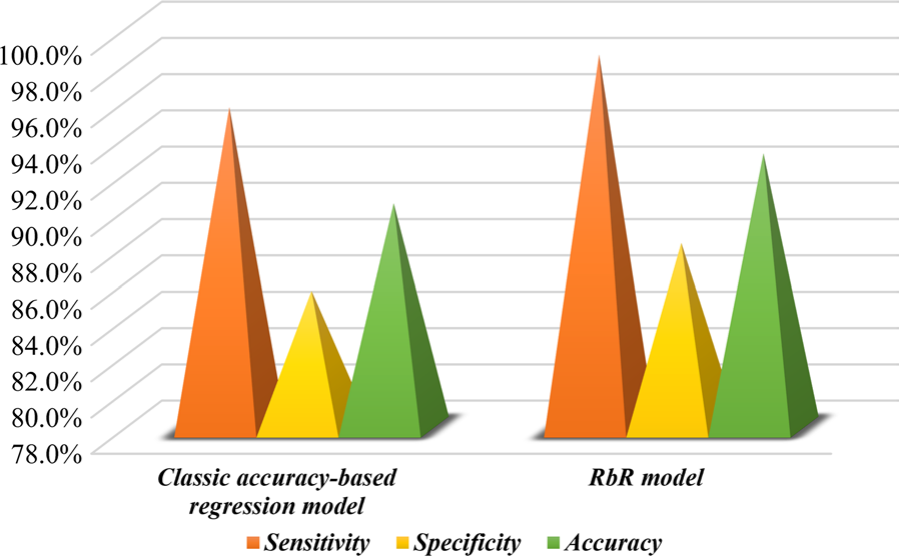
Comparison of performance of two proposed predictive models

**Fig. 2:**
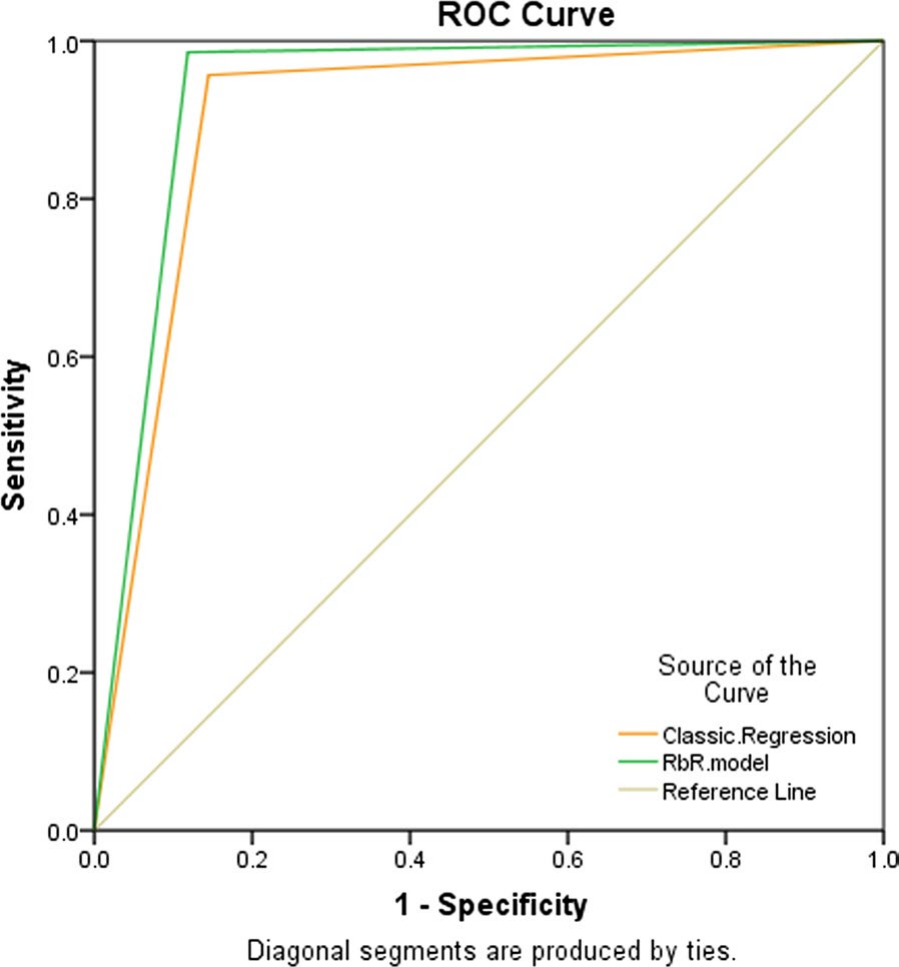
The ROC curves of proposed models

To assess the performance of the presented model, it is compared with the traditional regression model according to accuracy metric, i.e., the ratio of correctly predicted samples to the total number of samples. The results achieved by the proposed RbR and the classic regression models have been provided in Table 5 and Fig. 1. The performance results demonstrate that the proposed reliability-based approach, by yielding 98.6% sensitivity, 88.2% specificity, and 93.10% accuracy, has higher efficiency than its accuracy-based rival and even can successfully predict severe Covid-19 patients with more validity. Therefore, the proposed RbR model has provided more accurate results in distinguishing between the severe and mild cases of Covid-19 patients. Also, the graphical analysis of the ROC curve in Fig. 2 and its analysis in Table 6 shows that the proposed RbR model with a higher area under the curve (AUC) has a better performance than the classic regression model. The empirical results illustrate the importance of considering the reliability in predicting disease severity in Covid-19 patients and are important from two aspects. First, the proposed model can guarantee the reliability of predictions, especially in medical decision makings, which require stable and reliable results rather than accurate, because this model minimizes performance fluctuations. Secondly, the results show that the proposed reliability-based approach not only increases the reliability and stability of the results in medical decisions but also presents more accurate results than the classical accuracy-based regression method. Hence, the proposed RbR model not only solves the problem of unreliable results in traditional accuracy-based models, but also improves the accuracy of such models, so it can be a useful alternative for classic prediction models to adopt reliable and accurate medical decisions.

**Table 5:** Comparison of performance of proposed models

Models	Evaluation metrics		
	Sensitivity (%)	Specificity (%)	Accuracy (%)
Classic regression model	95.70	85.50	90.30
RbR model	98.60	88.20	93.10

**Table 6:** The ROC analysis of proposed models

Model	AUC	95%CI	*p* Value
Classic regression model	0.906	0.851–960	0
RbR model	0.934	0.887–980	0

## Conclusion

The accuracy of the prediction models plays a critical role in forecasting the severity of Covid-19 disease, but it is not the only effective factor to judge the generalizability of the models. Certainly, the reliability and confidence of the accuracy is another crucial factor that must be considered in modeling and forecasting the severity of Covid-19 patients. In this study, we have proposed a novel modeling approach to consider and maximize the reliability of the accuracy in predicting the severity of Covid-19 patients. For this, the classic regression model as a fundamental and common statistical method in disease predictions is applied. To show the generalization power of the proposed RbR model, we have applied a real-world dataset. The results imply that the proposed approach has not only increased the reliability of the results, it has also provided logical results about effective factors on the severity of Covid-19 patients and has yielded more accurate results compared with the classic accuracy-based regression model. The main contribution of the paper is the mathematical formulation of the proposed model. It is then used to analyze and forecast the severity of COVID-19 patients. The results of the suggested RbR model show the importance of the reliability effect on the generalization power of the classic regression model. For future works, performing the RbR model on other datasets of the severity of Covid-19 patients is suggested. Also, the reliability-based approach can be implemented on other types of existing models including different statistical or artificial intelligence forecasting models.

## Data Availability

The dataset used and analysed during the current study is available publicly from the link provided and also the corresponding author.

## Abbreviations

AUC: Area under the curve
CHD: Coronary heart disease
CRP: C-reactive protein
CSS: Chest severity score
CT: Computed tomography
CVD: Cerebrovascular disease
GA: Genetic algorithm
LDA: Linear discriminant analysis
LSTM: Long short term memory
MLP: Multilayer perceptron
MLR: Multiple linear regression
MRMR: Minimum redundancy and maximum relevance
NLR: Neutrophil to lymphocyte ratio
OLS: Ordinary least square
PLR: Platelet to lymphocyte ratio
RbR: Reliability-based regression
RSS: Residual sum of squares
SII: Systematic immune-inflammation
SVM: Support vector machine
